# Finite Element Solutions for Magnetic Field Problems in Terfenol-D Transducers

**DOI:** 10.3390/s20102808

**Published:** 2020-05-15

**Authors:** Duo Teng, Yatian Li

**Affiliations:** 1 School of Marine Science and Technology, Northwestern Polytechnical University, Xi’an 710072, China; liyatian_nwpu@hotmail.com; 2 National Key Laboratory for Underwater Information Processing and Control, Xi’an 710072, China

**Keywords:** Terfenol-D transducer, finite element method, magnetostrictive, bias magnetic field, eddy current

## Abstract

An appropriate magnetic design helps ensure that the Terfenol-D (Terbium- Dysprosium-Iron alloy) rods in giant magnetostrictive transducers have the perfect magnetostriction ability. To determine the optimum Terfenol-D rod state, a segmented stack configuration comprised by the Terfenol-D rods and NdFeB (neodymium-iron-boron) permanent magnets is presented. The bias magnetic field distributions simulated through the finite element method indicate that the segmented stack configuration is one effective way to produce the desired bias magnetic field. Particularly for long stacks, establishing a majority of domain to satisfy the desired bias magnetic field range is feasible. On the other hand, the eddy current losses of Terfenol-D rods are also the crucial to their magnetostriction ability. To reduce eddy current losses, the configuration with digital slots in the Terfenol-D rods is presented. The induced eddy currents and the losses are estimated. The simulations reveal that the digital slots configuration decreases the eddy current losses by 78.5% compared to the same size Terfenol-D rod with only a hole. A Terfenol-D transducer prototype has been manufactured using a Terfenol-D rod with a mechanical prestress of about 10 MPa and a bias magnetic field of about 42 kA/m. Its maximum transmitting current response of 185.4 dB at 3.75 kHz indicates its practicability for application as an underwater projector.

## 1. Introduction

Transducers are devices that convert electrical energy into acoustical energy or vice versa [1]. The energy conversion, known as transduction, can be accomplished through various physical processes, e.g., electrostatic, magnetostatic, electrostriction, magnetostriction, piezoelectricity, etc. [2]. Transducers utilize special physical effects, appropriate vibration systems, and indispensable accessory components to achieve the transduction. Unlike other electroacoustic devices, piezoelectric transducers and magnetostrictive transducers have the advantages of outstanding mechanoacoustical performance, reliable configurations, and convenient operation [3,4,5,6]. Therefore, these types of transducers are widely used in detection, prospecting, medical, and underwater acoustic applications [7]. Piezoelectric transducers utilize the piezoelectric effect, and their active materials include quartz, Rochelle salt, piezoceramic, etc. [8], while the magnetostrictive transducers utilize the magnetostrictive effect, and their active materials include nickel, nonmetallic ferrites, rare earth-iron compounds, etc. [9]. As a result of the development of the giant magnetostrictive material called Terfenol-D, the magnetomechanical properties of magnetostrictive transducers now surpass piezoelectric ceramics in some respects [10]. The advantages of Terfenol-D make it appropriate for use in actuators, motors, and transducers [11], where Terfenol-D transducers are also called giant magnetostrictive transducers. Terfenol-D transducers have attracted much interest because of their exceptional electroacoustic performance—particularly their high power capabilities [12]—which facilitates the development of projectors [13]. In 1977, Meeks and Timme investigated the advantages and disadvantages of the rare earth iron material, and developed a low-frequency, resonant, longitudinally vibrating piston-type rare earth iron underwater sound transducer [14]. In 2001, Butler presented a 2.5 kHz magnetostrictive sonar transducer with a maximum source level of 214.6 dB and examined the prestress and magnetic bias [15]. In 2016, Chai and Mo presented a gourd transducer driven by Terfenol-D rod and some of their simulations predicted high power and wide band performance [16]. With the continuous improvement and optimization of transducer configurations over the past four decades, giant magnetostrictive materials have been used as active cores for various types of transducers, e.g., Tonpilz type, flextensional type, hybrid type, and so on. As Terfenol-D transducers have developed, design and analysis methods have also improved. In 2000, Mo and Zhu employed ANSYS software to design a 14 kHz Terfenol-D longitudinal transducer, and the calculated results were in good agreement with measurements [17]. In 2005, Kim and Jung presented a finite element analysis for a Tonpilz magnetostrictive transducer by taking into account the nonlinear behavior of the magnetostrictive material and fluid interaction [18]. Many studies have discussed design and analysis methods, focusing on the electroacoustic characteristics of Terfenol-D transducers. Such implementations presuppose that the properties of Terfenol-D are unchangeable. However, Terfenol-D is known to be nonlinear and its performance is highly dependent on mechanical prestress and magnetic bias states [19]. Hence, determining how to obtain the appropriate Terfenol-D state is very important in designing Terfenol-D transducers. In particular, magnetic field problems related to the magnetostrictive material are among the key challenges [20]. In 1995, Benbouzid built a finite element model to optimize the design of magnetic circuits, including magnetostrictive rods [21]. In 2015, Sheykholeslami et al. designed a multi-resonance transducer using Terfenol-D, and the magnetic circuit to minimize the flux leakage was simulated by finite element method [22]. In 2015, Talebian et al. studied the classical eddy currents losses of Terfenol-D, and investigated the effects of magnetic field frequency, peak of magnetic flux density and diameter of Terfenol-D on the eddy currents losses [23]. In 2020, Huang et al. developed the magnetoelastic dynamic strain model for Terfenol-D transducers, which considered eddy current losses [24]. These previous studies provide insight into the design or analysis of Terfenol-D transducers from mechanical, magnetic, electrical, and thermal perspectives, respectively. 

This paper discusses the magnetic field problem in a Terfenol-D transducer, addressing both the effective configuration to produce the appropriate bias magnetic field within the Terfenol-D rods, and the feasible to reduce the eddy current losses of Terfenol-D rods.

## 2. Configuration of Terfenol-D Transducers

Terfenol-D transducers have sophisticated configurations. This is likely a result of the complicated conversions among electrical, magnetic, elastic, and acoustic energy that occur in these transducers. Outwardly, Terfenol-D transducers appear to achieve the conversion between electric and acoustic energy, while essentially achieving the conversion of magnetic and elastic energy. If classified according to function, the key components of Terfenol-D transducers include active cores, vibration systems, prestress systems, bias magnetic field systems, and alternating magnetic field systems. Of course, it is possible for certain parts to be shared among the above five components. For instance, the Terfenol-D core participates in every section. 

**Figure 1:**
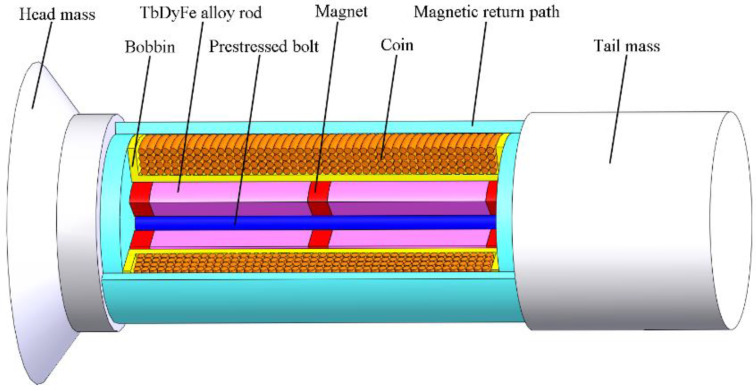
Schematic diagram of a Terfenol-D transducer.

The Figure 1 illustrates the configuration of a typical longitudinal magnetostrictive transducer, and Table 1 lists the transducer’s details. Its active core is the Terfenol-D rod with a hole. A prestressed bolt through the hole tightly connects to the radiating mass and tail mass. The static compression is provided to the Terfenol-D rod. This section not only achieves the prestress adjustment, but also functions as the main part of vibration. Many analyses have focused on this mechanical section, aiming to predict the electroacoustic performance of Terfenol-D transducers [25]. 

The transducer also has the magnetic section, which is relevant to the magnetic field problem in Terfenol-D transducers. A coil wraps directly around the Terfenol-D rod. The magnetic return path and the Terfenol-D rod comprise the magnetic circuit. Generally, some permanent magnets should be incorporated into the magnetic circuit to ensure the Terfenol-D rod obtains a given bias magnetic field when efficiency, limited power supply, temperature rise, continuous operation, etc. must be taken into consideration. Hence, the magnetic section related to the magnetic field problems is both important and difficult.

When Terfenol-D transducers operate as projectors, conversion based on the electric-magnetic-elastic-acoustic order is implemented. During the first stage, the excitation current flowing in the coil generates the desired alternating magnetic field within the Terfenol-D rod. This process will not achieve high efficiency without the appropriate magnetic circuit. In addition, methods for implementing the bias magnetic field in Terfenol-D rod and approaches to reducing the eddy current losses require consideration. All the magnetic field problems affect the performance of Terfenol-D transducers.

## 3. Finite Element Solution for the Magnetic Field Problem

Thus far, researchers have identified several effective methods for solving magnetic field problems, including the Ohm’s law method [26], the magnetic field division method [27], and the numerical method [28]. However, the finite element method has been shown to have incomparable advantages in electromagnetic field analysis [29]. Its basis is Maxwell’s equations, the differential forms of which are given by

(1) $$\begin{array}{c} \nabla \times H=J+\frac{\partial D}{\partial t}\\ \nabla \times E=-\frac{\partial B}{\partial t}\\ \nabla \cdot B=0\\ \nabla \cdot D=q \end{array}$$

where **E** is the electric field vector, **D** is the electric flux density or electric displacement vector, **H** is the magnetic field vector, **B** is the magnetic flux density vector, **J** is the volumetric current density vector, and q is the electric free charge density. 

The magnetic field problems in Terfenol-D transducers should belong to the quasi-stationary magnetic field domain because the operating frequencies of the Terfenol-D transducers are very low. When the time rate of change (frequency) of the driving source is slow, the field will become a quasistatic field [30]. Neglecting the displacement currents (quasi-stationary limit), Maxwell’s equations are given by:

(2) $$\begin{array}{c} \nabla \times H=J\\ \nabla \times E=-\frac{\partial B}{\partial t}\\ \nabla \cdot B=0 \end{array}$$

Moreover, the usual constitutive equations for magnetic and electric fields are:

(3) J=σB

(4) H=μB

where **σ** is the electrical conductivity matrix and **μ** is the magnetic permeability matrix.

In practice, solving the above equations is inconvenient. An effective method is to introduce the potential functions. The two kinds of potential functions include the magnetic vector potential and the magnetic scalar potential [31]. The choice of the most appropriate formulation depends on the problem to be solved and the factors that affect such choices include field dynamics, field dimensionality, source current configuration, domain size, and discretization [32].

The magnetic vector potential, **A,** and the electric scalar potential, *V*, satisfy the link with the magnetic flux density, **B,** and the electric field, **E,** as follows:

(5) B=∇×A

(6) $$E=-\frac{\partial A}{\partial t}-\nabla V$$

The magnetic vector potential, **A**, is a useful computing tool. It is the primary quantity obtained via FEM. The other magnetic field quantities are calculated subsequently, e.g., magnetic field flux density, current density, energy, loss, etc. [33].

## 4. Bias Magnetic Field within the Terfenol-D Rod and the Magnetic Circuit of the Terfenol-D Transducer

As is well known, if a Terfenol-D rod is excited sinusoidally without any bias magnetization, the frequency of the strain response will be double the excitation frequency. Avoiding this phenomenon requires an appropriate bias magnetostriction that is at least larger in magnitude than the amplitude of the dynamic field. Considering the strain response as linear as possible, it is better to locate within the region where the response is significant and approximately linear. 

Two factors affect the optimum state of Terfenol-D rods. One is the prestress, which is achieved in the mechanical prestress system. In our design, the prestress of compressing the Terfenol-D rod is 10 MPa [34]. The other is the bias magnetostriction, which is generated in the magnetic system. In our design, the bias Magnetic Field Intensity (bias MFI) within the Terfenol-D rod should be approximately 42 kA/m [34]. A Terfenol-D rod with the appropriate prestress and bias MFI will exhibit a near linear behavior and provide the desired magnetostriction effect so long as the total magnetic field including the sinusoidal excitation is kept within a reasonable range, at least below the saturation.

**Figure 2:**
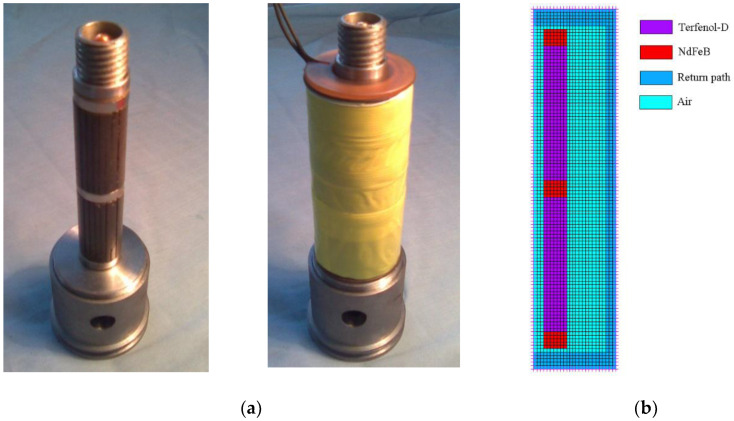
Stacked core of the Terfenol-D transducer and the finite element model of the magnetic circuit. (**a**) The segmented stack; (**b**) the finite element model.

Figure 2a shows the core components of the Terfenol-D transducer. It is a stack that includes two pieces of Terfenol-D rods and three pieces of NdFeB permanent magnets one by one in mechanical series. We designed such a segmented stack with more than one Terfenol-D rod to obtain the desired bias magnetic field. The Terfenol-D rod is excited by an AC driving coil. In addition, a flux return path with high permeability (DT4E grade electromagnetism pure iron) is added. All the above components comprise the magnetic circuit. Among the components, we focus on the Terfenol-D rods, especially their magnetic fields, which are generated by the NdFeB magnets with a coercive force of 935 kA/m in our design. Because the permanent magnets must be considered, the constitutive relationship for the magnetic fields becomes:

(7) $$B=\mu H+\mu_{0}M_{0}$$

where $\mu_{0}$ is the permeability of free space and $M_{0}$ is the remanent intrinsic magnetization vector.

**Figure 3:**
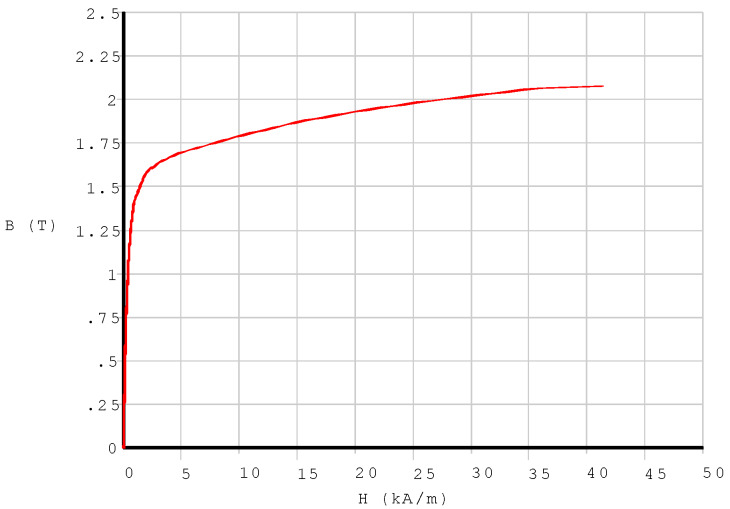
The nonlinear B-H curve of the electromagnetism pure iron used as magnetic return path.

During modeling, the magnetic permeability of the above components should be specified. To simplify the modelization, the assumption of linearity for some material properties will be always permitted, because Terfenol-D transducers are actually always restricted to operating in the range of approximate linearity, even though Terfenol-D’s magnetostriction effect is nonlinear [35,36]. Table 2 displays the material properties of the components in the magnet circuit. However, the iron is special; its nonlinear properties of B-H must be input (Figure 3).

Figure 2b presents the finite element model of the magnetic circuit. Considering the convenience of modeling and the solution speed, a two-dimensional (2D) axisymmetric model has been built because the actual magnetic circuit is symmetrical around the axis. During the finite element simulation, the 2D solid element type PLANE13, which uses the magnetic vector potential formulation, is available for modeling the magnet circuit. The special physics attributes of each region in the model should be assigned as shown in Table 2. Boundary conditions and loads (excitation) are needed, and the magnetic solution options should be specified. After the implementation of the solution, the results, including the primary data, the magnetic vector potential, and the derived data (the magnetic flux density, the magnetic field intensity, etc.) are obtained.

**Figure 4:**
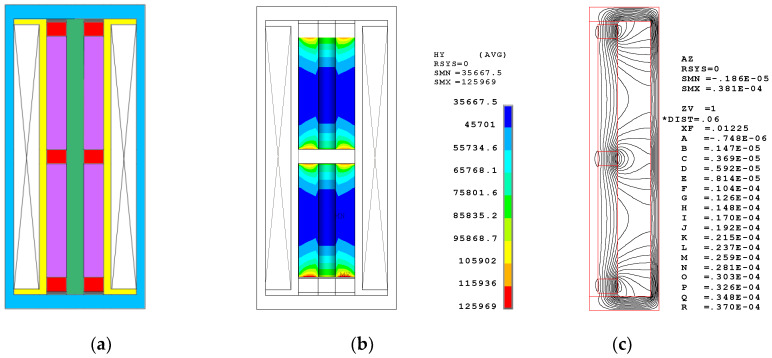
Segmented stack configuration and the finite element simulations. (**a**) The configuration; (**b**) the Bias Magnetic Field Intensity (MFI) distribution; (**c**) the Flux distribution.

Figure 4 shows the configuration, the bias magnetic field distribution in the Terfenol-D rod, and the contour flux line plot in the magnetic circuit. The segmented stack includes two pieces of the Terfenol-D rods with sizes of (φ20 × φ6 × 40) mm and three pieces of NdFeB permanent magnets with sizes of (φ20 × φ6 × 5) mm. Figure 4b shows the contour distribution of the bias magnetic field in the Terfenol-D rod. Table 3 displays the details. Within the Terfenol-D rod, the mean MFI is approximately 56 kA/m. Figure 7 illustrates the volumetric proportion of the bias magnetic field within the Terfenol-D rod, which intuitionally shows the distribution of the bias MFI. The covered region of every curve is equal to 100%. From a practical engineering perspective, it is impossible for the Terfenol-D rod to obtain a constant bias MFI of, for instance, 42 kA/m, by way of using permanent magnets. This causes the bias magnetic field to be distributed in a certain range. The concentration of the generated magnetic field at approximately 42 kA/m is the only desired object. In this segmented stack configuration, about 80.54% of the domain obtains a bias MFI from 35 to 70 kA/m, which is the range necessary to ensure that Terfenol-D rods work. The contour distribution shows that the max MFI occurs in the domain near the permanent magnets, while the min MFI occurs in the middle domain. The distribution shows a standard deviation of 20,835. Figure 4c shows the contour flux line plot in the magnetic circuit. The flux flows approximately parallel to the Terfenol-D rod. In addition, a certain amount of undesirable flux leakage occurs in the open domain.

**Figure 5:**
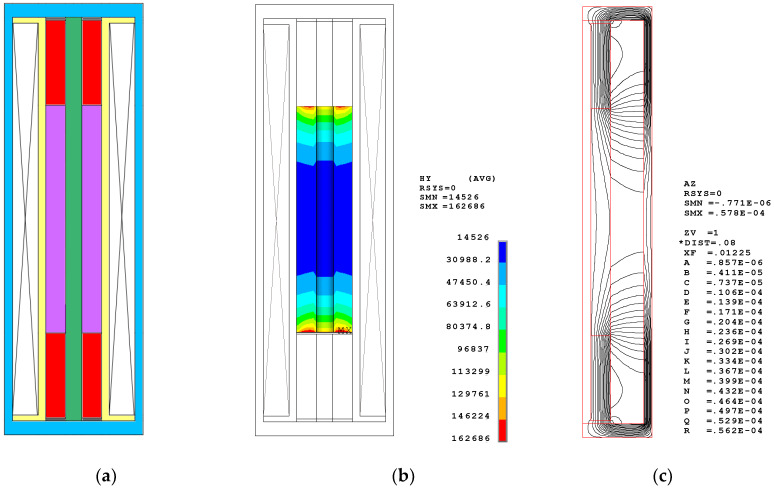
Nonsegmented configuration and the finite element simulations. (**a**) The configuration; (**b**) the Bias Magnetic Field Intensity (MFI) distribution; (**c**) the Flux distribution.

To obtain an intuitional comparison, the nonsegmented configuration and DC coil configuration, in which the total length of Terfenol-D rod is the same, are also simulated. The nonsegmented configuration includes only one Terfenol-D rod with a size of (φ20 × φ6 × 80) mm and two pieces of NdFeB permanent magnets with sizes of (φ20 × φ6 × 30) mm. Figure 5a shows the schematic configuration. The bias magnetic field distributes in a large range from 14 to 160 kA/m. As the details in Table 3 show, the mean MFI is about 46 kA/m. Only 26.43% of the domain satisfies the desired bias MFI of 35–70 kA/m. More than half of the domains have bias magnetic fields below 35 kA/m. At the same time, the standard deviation of the nonsegmented configuration is 34,520—larger than the segmented stack configuration. This index has a dissatisfactory concentration. Obviously, this distribution will hamper the efficiency of Terfenol-D because too low bias magnetization causes it to diverge from its appropriate state. In this simulation, the total length of the two permanent magnets is 60 mm. This is also too long compared to the Terfenol-D rod. Therefore, continuous elongation of the permanent magnets is an ineffective way to obtain the desired bias magnetic field. In addition, the flux distribution reveals nonparallel magnetization and more flux leakage.

**Figure 6:**
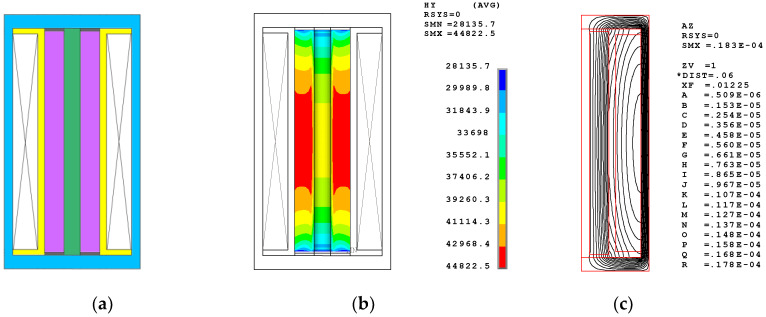
DC driving coil configuration and the finite element simulation. (**a**) The configuration; (**b**) the Bias Magnetic Field Intensity (MFI) distribution; (**c**) the Flux distribution.

The DC coil configuration shown in Figure 6 is designed to obtain the bias magnetic field using one DC solenoid coil. In such a nonpermanent magnetic circuit, a direct current of at least 5 amperes and a coil with at least 682 winding turns are necessary to obtain the bias MFI of 42 kA/m within a Terfenol-D rod with a length of 80 mm. Such a DC coil produces a mean MFI of about 41 kA/m. More than 91% of the domain satisfies the desired bias MFI of 35–50 kA/m and is located in the middle domain. The standard deviation is only 3814, which is the smallest value among the three configurations. Moreover, the flux lines within the Terfenol-D rod are almost parallel. All of the distributions demonstrate the uniformity and centrality of bias magnetic field. However, the heat problem is a critical defect. A simple test shows that it takes only two or three minutes for the coil to exceed 80 °C when the driving DC is 5 amperes. Generating so much heat in the application is highly problematic. In addition, an extra DC coil will increase the complexity of the transducer configuration.

**Figure 7:**
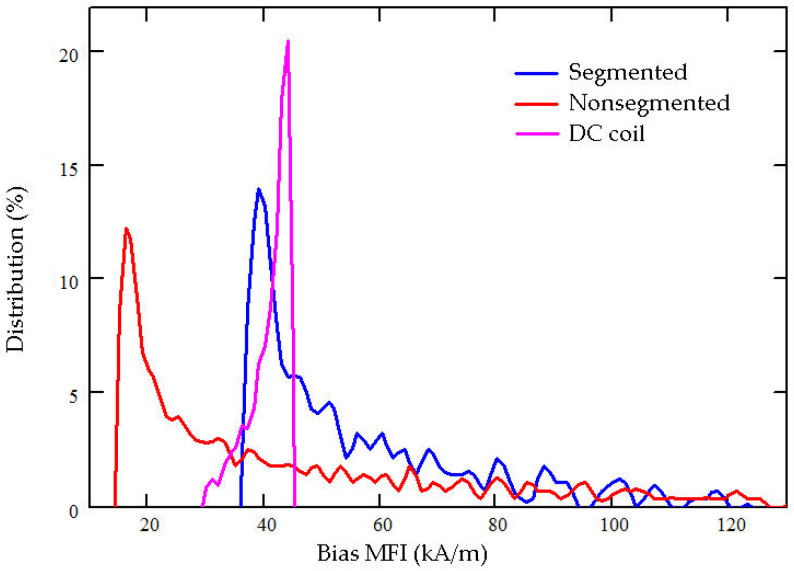
Bias magnetic field distribution within the Terfenol-D rod for three configurations.

How the bias magnetic field within the Terfenol-D rod is obtained depends on either the permanent magnets or the DC coil. Estimating the advantages and disadvantages of the different methods is crucial. The curves shown in Figure 7 indicate that the bias magnetic fields generated by using permanent magnets cover the range from low field to 126 kA/m. The high fields occur near the permanent magnets, shown in Figure 4 and Figure 5, and are unavoidable as long as permanent magnets are used. This is not a fatal flaw because the high field proportion is very low. 

Our comparison of the above finite element simulations reveals the characteristics of various solutions. The basic premise is the same size of Terfenol-D rod in total. The DC coil configuration has advantages when it comes to obtaining a uniform bias magnetic field, where it has the best comparative quality, but it suffers the disadvantage of generating more heat. The segmented stack configuration makes it easy to produce the bias magnetic field with a medium level of quality. The nonsegmented configuration produces a terrible bias magnetic field when the Terfenol-D rod has a length of 80 mm. However, it is suitable when the Terfenol-D rod and the permanent magnets are of appropriate sizes, and a majority of the domain within Terfenol-D rod satisfies the desired range of the bias magnetic field.

## 5. Eddy Current Losses of the Terfenol-D Rod in the Terfenol-D Transducer

The eddy current losses are unavoidable in applying magnetostrictive transducers. When the Terfenol-D rod is located in an alternating magnetic field, which is produced by an alternating driving current flowing in the surrounding coil, an induced current will occur in the rod. This current will cause power losses due to the electrical resistivity of the magnetostrictive material. It will also generate another magnetic field to oppose the primary magnetic field produced by the coil. Generally, the distribution of the eddy current is described by the skin depth, δ, which is defined as the depth below the surface of a conductor where the current density has been reduced to 1/e times its value at the surface of the conductor:

(8) $$\delta =\sqrt{\frac{1}{\mu \sigma \pi f}}$$

where *σ* is the electrical conductivity, *μ* is the magnetic permeability, and *f* is the frequency.

Terfenol-D is a conductor with a resistivity of 0.6 μΩm [37] and the eddy current will inevitably occur. Hence, reducing the eddy current losses is very important in designing Terfenol-D transducers. Three factors affect the eddy current within the Terfenol-D rod—the frequency of the driving magnetic field, the resistivity, and the shape, where the shape is optimizable. Laminating the Terfenol-D rod is an effective way to reduce eddy current losses, but doing so involves the difficult step of gluing the laminated layers together with an epoxy adhesive. In our design, a Terfenol-D rod with digital slots is developed, and its eddy current losses are simulated through FEM. 

**Figure 8:**
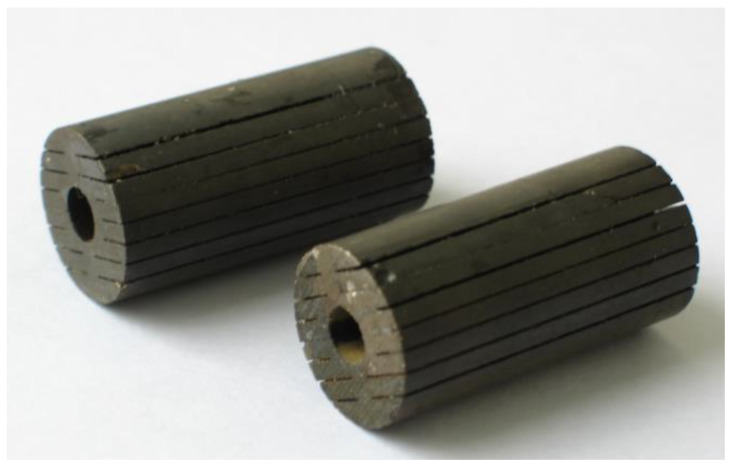
The Terfenol-D rod with digital slots.

**Figure 9:**
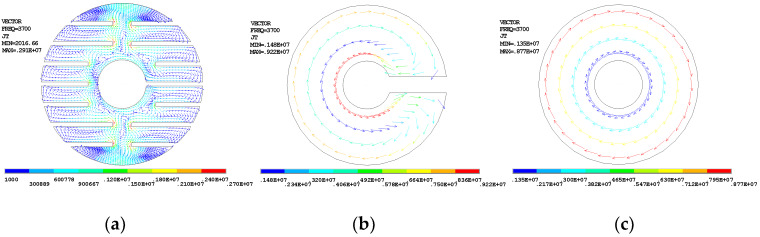
The eddy current distribution for three different rod shapes. (**a**) Rod with digital slots; (**b**) rod with a single slot; (**c**) rod with a hole.

Figure 8 shows the Terfenol-D rod with digital slots. Its size is (φ20 × φ6 × 40) mm. All the slots are processed by line cutting, and are entirely filled with the epoxy resin. There is a hole at the center, through which a prestressed bolt passes. Effectively eliminating these losses requires the use of the finite element model to define two or three elements in the thickness according to the skin depth [38]. Three Terfenol-D rods with different cross-section shapes are simulated. They are the rod with digital slots, the rod with a single slot, and the rod with a hole, respectively. Figure 9 shows the eddy current distributions, which are generated in a coil with 682 winding turns, an alternating current of 5 amperes, and an operation frequency of 3.7 kHz. All the distributions show that the eddy current flowing near the surface is larger, which aligns with the skin effect. Among the three configurations, the loop of the rod with digital slots is the longest, while the current density in this rod is very small. The rod with a single slot and the rod with a hole have the same loop thickness, but these rods have different loop lengths. The above rod shapes cause different eddy current losses. Using the rod with a hole as reference, the time-averaged eddy current losses of the rod with a single slot increased by 20%, while those of the rod with digital slots decreased by 78.5%. That digital slotting reduces the losses is thus a very obvious effect.

## 6. Prototypes and Tests

**Figure 10:**
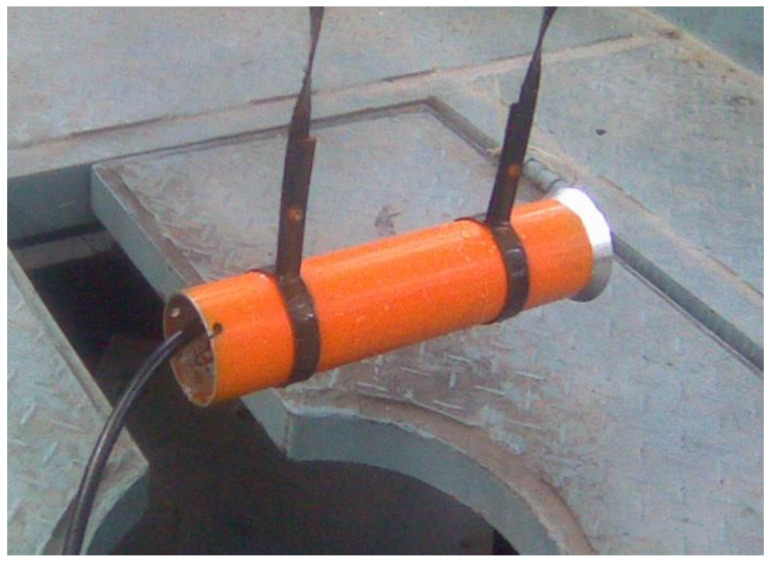
The Terfenol-D transducer prototype.

**Figure 11:**
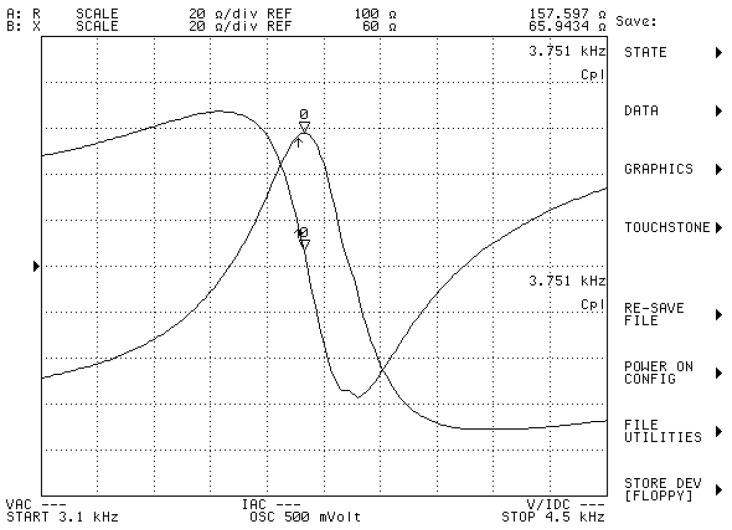
The impedance curves of the Terfenol-D transducer in water (obtained via Agilent 4294A).

Figure 10 shows a Terfenol-D transducer prototype, which is manufactured according to the magnetic design outlined above. Its impedance curves are obtained using the precision impedance analyzer Agilent 4294A (Agilent, Santa Clara, CA, USA). When the transducer is submerged in water and tested, its resistance curve peaks at a frequency of 3.75 kHz, as shown in Figure 11. The reactance is inductive—a marked difference from the capacitive property of piezoelectric transducer. As an underwater projector, its electroacoustic characteristics are measured in the anechoic tank. When the Terfenol-D transducer is submerged and is driven by an alternating current, the acoustic pressure is generated in the water. The pressure variation is detected by a calibration hydrophone at a distance in the far field. The Transmitting Current Response (TCR), the response per ampere, describes the transmitting characteristics of the projector. Our Terfenol-D transducer presents a maximum transmitting current response (TCR) of 185.4 dB at the resonance frequency. The appropriate state of the Terfenol-D rod and the use of an effective method for reducing the eddy current losses facilitate this high TCR. The mechanical prestress of about 10 MPa and the bias MFI of about 42 kA/m are suitable for the Terfenol-D rod with digital slots in the application of projectors. 

## 7. Conclusions

This paper presents a Terfenol-D transducer utilizing the Terfenol-D rods and discusses its magnetic design. The distributions of the bias magnetic field within the Terfenol-D rod, the flux line in the magnetic circuit, and the eddy current induced within the Terfenol-D rod are estimated using the finite element method and the relevant electroacoustic tests are conducted. In summary, the following conclusions can be drawn.

(1). Three different configurations are simulated and analyzed to estimate the effect of generating the bias magnetic field within the Terfenol-D rod and the result of distributing the flux line along the magnetic circuit. A comparison of the simulation results indicates that the segmented stack configuration is the most effective way to produce the desired bias magnetic field. Especially for the long stack, making a majority of the domain satisfy the desired bias magnetic field range is achievable. When adopting this approach as a guideline, the nonsegmented configuration will also work so long as the Terfenol-D rod is appropriately short.
(2). Three different configurations are simulated and analyzed to estimate the eddy current losses within the Terfenol-D rod. A comparison of the simulation results indicates that the configuration with digital slots is the most effective way to reduce the losses. It decreases the eddy current losses by 78.5% compared to a Terfenol-D rod of the same size with a single hole.
(3). When the mechanical prestress is about 10 MPa and the bias magnetic field intensity is about 42 kA/m, the Terfenol-D rod will achieve an appropriate state and make the application of underwater projectors highly efficient.
(4). Using the finite element method with the magnetic vector potential is an effective way to simulate magnetic field problems in Terfenol-D transducers. Its intuitional displays and rapid solutions help facilitate magnetic design of Terfenol-D transducers.

## Figures and Tables

**Table 1:** The materials and sizes of the Terfenol-D transducer’s main components.

Components	Materials	Description	Quantity
Head mass	Aluminum alloy	Radiating surface diameter	φ70 mm
		Laryngeal diameter	φ54 mm
		Length	20 mm
Magnetostrictive rod	Terfenol-D	Outer diameter	φ20 mm
		Inner diameter	φ6 mm
		Length of single piece	40 mm
		Pieces	2
Permanent magnets	NdFeB	Outer diameter	φ20 mm
		Inner diameter	φ6 mm
		Length of single piece	5 mm
		Pieces	3
Coil	Enameled copper wire	Turns	682
		Wire diameter	1 mm
Magnetic return path	Electromagnetism pure iron	Outer diameter of cylinder	φ49 mm
		Inner diameter of cylinder	φ43 mm
		Length of single piece	97 mm
		Diameter of shields	φ49 mm
		Length of shields	5 mm
		Pieces of shields	2
Prestressed bolt	Nonmagnetic steel	Size	M5
Tail mass	Nonmagnetic steel	Diameter	φ54 mm
		Length	55 mm

**Table 2:** Material properties of the components in the magnet circuit.

Components	Materials	Property
Magnetostrictive rod	Terfenol-D	Relative permeability: 5
Permanent magnets	NdFeB	Relative permeability: 1.14
		Coercive force: 935 kA/m
Magnetic return path	Electromagnetism pure iron	B-H curve (See Figure 3)
Other sections	Air	Relative permeability: 1

**Table 3:** Statistical quantities for the three configurations.

Quantity	Standard Deviation	Mean MFI (A/m)	Min MFI (A/m)	Max MFI (A/m)	Terfenol-D Size (mm)	NdFeB Size (mm)
Segmented	20,835	56,112	35,668	125,969	(φ20 × φ6 × 40)	(φ20 × φ6 × 5)
					× 2 pcs	× 3 pcs
Nonsegmented	34,520	46,105	14,526	162,686	(φ20 × φ6 × 80)	(φ20 × φ6 × 30)
					× 1 pcs	× 2 pcs
DC coil	3814	40,566	28,136	44,822	(φ20 × φ6 × 80)	
					× 1 pcs	
